# Enhancing cardiometabolic health: unveiling the synergistic effects of high-intensity interval training with spirulina supplementation on selected adipokines, insulin resistance, and anthropometric indices in obese males

**DOI:** 10.1186/s12986-024-00785-0

**Published:** 2024-03-07

**Authors:** Maryam Delfan, Ayoub Saeidi, Rashmi Supriya, Kurt A Escobar, Ismail Laher, Katie M. Heinrich, Katja Weiss, Beat Knechtle, Hassane Zouhal

**Affiliations:** 1 Department of Exercise Physiology, Faculty of Sport Sciences, Alzahra University, Tehran, Iran; 2 Department of Physical Education and Sport Sciences, Faculty of Humanities and Social Sciences, University of Kurdistan, Sanandaj, Kurdistan Iran; 3 Centre for Health and Exercise Science Research, SPEH, Hong Kong Baptist University, Kowloon Tong, Hong Kong SAR China; 4 Department of Kinesiology, California State University, Long Beach, CA 90840 USA; 5 Department of Anesthesiology, Pharmacology, and Therapeutics, Faculty of Medicine, University of British Columbia, Vancouver, Canada; 6 Department of Kinesiology, Kansas State University, Manhattan, KS 66502 USA; 7 Institute of Primary Care, University of Zurich, Zurich, Switzerland; 8 Medbase St. Gallen Am Vadianplatz, Vadianstrasse 26, St. Gallen, 9001 Switzerland; 9 Univ Rennes, M2S (Laboratoire Mouvement, Sport, Rennes, Santé EA 1274, F-35000 France; 10 Institut International des Sciences du Sport (2I2S), Irodouer, 35850 France

**Keywords:** Spirulina, High-intensity interval training, Adipokines, Obesity

## Abstract

This study investigated the combined effects of 12 weeks of high-intensity interval training (HIIT) and spirulina supplementation on adipokine levels, insulin resistance, anthropometric indices, and cardiorespiratory fitness in 44 obese males (aged 25–40 years). The participants were randomly assigned to one of four groups: control (CG), supplement (SG), training (TG), or training plus supplement (TSG). The intervention involved daily administration of either spirulina or a placebo and HIIT three times a week for the training groups. Anthropometric indices, HOMA-IR, VO_2peak_, and circulating adipokines (asprosin and lipocalin2, omentin-1, irisin, and spexin) were measured before and after the 12-week intervention. Post-intervention analysis indicated differences between the CG and the three interventional groups for body weight, fat-free mass (FFM), percent body fat (%BF), HOMA-IR, and adipokine levels (*p* < 0.05). TG and SG participants had increased VO_2peak_ (*p* < 0.05). Spirulina supplementation with HIIT increased VO_2peak_, omentin-1, irisin, and spexin, while causing decreases in lipocalin-2 and asprosin levels and improvements in body composition (weight, %fat), BMI, and HOMA-IR. Notably, the combination of spirulina and HIIT produced more significant changes in circulating adipokines and cardiometabolic health in obese males compared to either supplementation or HIIT alone (*p* < 0.05). These findings highlight the synergistic benefits of combining spirulina supplementation with HIIT, showcasing their potential in improving various health parameters and addressing obesity-related concerns in a comprehensive manner.

## Introduction

Overweight and obesity describe the accumulation of excessive or abnormal fat, with potential health risks. Forecasts suggest that by 2035, more than half of the global population will grapple with overweight or obesity [1]. The incidence of obesity in Iran escalated from 2 million in 1980 to 11 million in 2015 [2]. Established guidelines from entities like the World Health Organization and the U.S. Department of Health and Human Services recommend that individuals partake in at least 150–300 min of moderate-intensity or 75–150 min of vigorous-intensity physical activity weekly [3]. Alongside traditional physical activity, high-intensity interval training (HIIT) is a promising avenue for altering body composition and triggering various metabolic responses [4–7]. For instance, supervised HIIT involving 30-second bursts at 90% VO_2max_ followed by 30-second recovery periods, conducted three times weekly for four weeks, enhanced fat oxidation by approximately 31% after a 30-minute constant load exercise session at 45% intensity in overweight and obese men [8].

Several studies [9–12] described the role of inflammation in obesity, highlighting novel adipokines such as asprosin, lipocalin2, omentin-1, irisin, and spexin. These adipokines play intricate roles in metabolic dysregulation, cardiovascular conditions, and the inflammatory state associated with obesity [9–12]. Serum levels of asprosin correlate positively with BMI, waist circumference, and glycemic and lipid parameters, particularly in individuals with abdominal obesity [13]. In contrast, lipocalin2 levels are approximately 60% higher in obese individuals [14]. The recently identified adipokine spexin, regarded as a regulator of obesity and related comorbidities, is reduced in obese patients [15]. Omentin-1, known for augmenting insulin-mediated glucose uptake, is implicated in insulin resistance [16, 17]. Similarly, irisin, a skeletal muscle glucose uptake regulator, improves hepatic glucose and lipid metabolism while mitigating obesity-related hyperlipidemia and hyperglycemia [18]. Irisin’s potential to enhance insulin sensitivity correlates with a lower incidence of type 2 diabetes mellitus [19, 20].

Moving beyond metabolic disturbances, chronic inflammation and systemic oxidative stress also contribute significantly to obesity and exacerbate cardiovascular dysfunction [21]. Spirulina, a cyanobacterium commonly known as blue-green algae, can potentially reduce various obesity-related parameters [22]. Studies, primarily conducted in women, indicate that spirulina elevates glutathione levels, fat oxidation, and exercise performance while reducing exercise-induced lipid peroxidation [23]. A combined approach involving spirulina supplementation (4.5 g per day for six weeks) and HIIT (performed thrice weekly) has beneficial effects on blood lipids and BMI in sedentary men with excessive body weight [24].

Our investigation aims to scrutinize the hypothesis that an extended administration of spirulina supplementation (over 12 weeks), coupled with HIIT, could potentially mitigate obesity and induce favorable alterations in the levels of pro-inflammatory adipokines (asprosin and lipocalin2) and anti-inflammatory adipokines (omentin-1, irisin, and spexin) within obese males.

## Methods

### Ethical approval

A written informed consent for study participation was obtained from all study participants before the study started. The study was approved by Sport Sciences Research Institute Ethics Committee of Alzahra University (Tehran, Iran). (Ethics code: IR.SSRC.REC.1401.093). All procedures were performed according to the latest revision of the Declaration of Helsinki [25].

### Participants

Following recruitment efforts in various settings, including public spaces, laboratories, sports clubs, and social networks, a total of 143 individuals volunteered to participate in the study, of which 80 were deemed ineligible for participation (aged 25–40 years). Inclusion criteria for participation were: a body mass index (BMI) exceeding 30 kg/m², a lack of engagement in regular physical activity during the preceding six months, no cardiovascular and endocrine disorders, and non-consumption of alcohol.

A subset of 80 individuals were carefully selected to participate in the study after an initial evaluation of volunteers. In order to meet inclusion criteria, all participants underwent a thorough physical examination conducted by a qualified medical professional and clinical exercise physiologist during the initial visit. Following the initial assessment and a detailed explanation of the research components, 64 individuals were ultimately chosen. The determination of the sample size was based on the standardized effect size (SES), calculated utilizing mean and standard deviation values from other comparable studies [26].

This SES was analyzed using the G*Power (3.1.9.4) program [two-sided, α = 0.05, power (1-β) = 0.95, effect size = 1.43], which indicated a minimum required sample participant size per group of nine. For this study, we opted to include 16 subjects in each group. Participants provided a signed consent form and completed the Physical Activity Readiness Questionnaire (PAR-Q) [27]. This procedural step was implemented to uphold compliance with established research standards and ethical guidelines.

### Experimental design

Before commencing the training programs, participants engaged in a familiarization session one week in advance, wherein a comprehensive explanation of all study procedures was provided. The participants’ height, mass, and body composition were measured. Following this, a random assignment process was undertaken, placing individuals into one of four equally sized groups: Control Group (CG), Supplement Group (SG), Training Group (TG), and Training + Supplement Group (TSG). Over the course of the study, 20 participants in different groups withdrew due to medical reasons, job-related constraints, and waning interest in the research. This attrition led to 11 participants remaining within each group for subsequent analysis.

All groups received instructions on the execution of the training protocols during the third session, which coincided with the measurement of body composition variables and peak oxygen uptake (VO_2peak_). The 12-week exercise training program of three sessions per week, was initiated in the two training groups (TG and TSG) after baseline assessments. In contrast, participants in the CG were instructed to maintain their existing lifestyles throughout the study.

To ensure consistency and minimize confounding factors, data collection occurred at identical times of day, typically within an hour window, and under uniform environmental conditions of ~ 20 °C and a relative humidity of ~ 55%. Baseline measurements were acquired 48 h before the onset of the training protocols, while post-tests were executed 48 h after the final session in all groups. Participants in the two training groups were instructed to maintain a consistent dietary regimen during the 48 h preceding the baseline assessment and the final measurements after training.

### Body composition and cardio-respiratory fitness assessments

Body weight and stature were gauged using a calibrated scale and stadiometer (Seca, Germany). These measurements were subsequently employed to compute BMI (kg/m²). Determination of fat-free mass (FFM) and fat mass (FM) was accomplished using a bio-impedance analyzer (Medigate Company Inc., Dan-dong Gunpo, Korea).

The evaluation of VO_2peak_ was conducted using a modified Bruce protocol in a controlled environment (21–23 °C), consistent with established practices for overweight and obese cohorts [28, 29]. We utilized an electronically motorized treadmill (H/P/Cosmos, Pulsar med 3p- Sports & Medical, Nussdorf-Traunstein Germany). Our approach followed the physiological criteria outlined in the American College of Sports Medicine (ACSM) guidelines. Participants were deemed to have achieved VO_2peak_ when indicating physical exhaustion and maximal exertion (according to the Borg scale) or if supervisory personnel identified severe dyspnea, dizziness, or other limiting symptoms, aligning with ACSM and American Heart Association (AHA) protocols [30, 31].

Blood pressure measurements were acquired utilizing an electronic sphygmomanometer (Kenz BPM AM 300P CE, Japan), while heart rate was continuously monitored using a Polar V800 heart monitor (Finland) throughout the testing sessions. Gas analysis was conducted with a gas analyzing system (Metalyzer 3B analyzer, Cortex: biophysics, GMbH, Germany) that was calibrated before each test.

### Training protocols

The exercise intensity during training sessions was based on VO_2peak_ values based on a 32-minute running exercise on a treadmill. Participants engaged in a 5-minute warm-up phase of stretching maneuvers, walking, and running prior to each training session. Participants performed treadmill running at an intensity set at 65% of their VO_2peak_ during the first week, which was then progressively increased to 75% of their VO_2peak_ in the subsequent week. The control of exercise intensities throughout the sessions was managed by adjusting the treadmill settings based on the specified percentages of VO_2peak_. This standardized approach was consistently maintained across three weekly sessions.

Transitioning into the third week marked the commencement of High-Intensity Interval Training (HIIT) sessions. Over the third and fourth weeks, participants executed intervals comprising 4 min of running at 75% of their VO_2peak_, followed by 4 min of passive recovery, for a 32-minute duration of HIIT. In weeks 5, 6, and 7, intervals were extended to 4 min of running at 85% of their VO_2peak_, interspersed with 4-minute active recovery intervals at 15% of their VO_2peak_, maintaining the 32-minute HIIT duration. For weeks 8, 9, and 10, participants engaged in 4-minute intervals at 90% of their VO_2peak_ and active recovery intervals at 30% of their VO_2peak_ for 4 min, all executed on the treadmill within the 32-minute timeframe. The final stretch (weeks 11 and 12) consisted of 4-minute intervals of running at 95% of their VO_2peak_ and 4-minute intervals of active rest at 50% of their VO_2peak_ for 32 min. Participants performed a 5-minute cool-down at 50% of their VO_2peak_ after each training session [32]. In contrast, the control group continued their customary daily activities and did not engage in routine physical exercise.

### Supplementation of spirulina and placebo

Spirulina (Hellenic Spirulina Net. Production unit: Thermopigi, Sidorokastro, Serres, Greece) was prepared as capsules for administration. Each subject ingested a daily dosage of 6 g of spirulina, divided into two equal portions of three grams each, taken in the morning and evening, for 12 weeks [33]. Both the CG and the TG were also provided with an equivalent quantity of placebo. This placebo was constituted of corn starch, which was appropriately colored with edible green dye to mimic the appearance of spirulina powder, and further flavored with essence of kiwi fruit. Participants’ adherence to the supplement regimen was considered satisfactory when consumption equaled or exceeded 80% of the stipulated supplements.

### Nutrient intake and dietary analysis

Three-day dietary records were procured, encompassing two weekdays and one weekend day, before and after the study. These records monitored alterations in customary dietary patterns over the study duration [34]. Each specific food item was entered in the Diet Analysis Plus version 10 software (Cengage, Boston, MA, USA), which calculated the total caloric intake and the proportion of energy derived from proteins, fats, and carbohydrates (Table 1).

**Table 1:** Mean (± SD) values of nutritional intake in the four study groups

	**CG**		**SG**		TG		**TSG**	
	Pre	Post	Pre	Post	Pre	Post	Pre	Post
Energy (kcal/d)	2321 ± 47	2342 ± 56	2354 ± 101	2314 ± 100	2349 ± 117	2297 ± 117	2375 ± 157	2301 ± 126
CHO (g/d)	292 ± 30.4	295 ± 31.3	288.4 ± 25.1	278 ± 26.5	298 ± 41.6	270 ± 37.2	297 ± 39.6	269 ± 30.1
Fat (g/d)	91.2 ± 16.0	92 ± 19.8	95.5 ± 17.7	84 ± 16.2	94.4 ± 19.4	84.1 ± 15.2	918 ± 15.87	75.2 ± 18.3
Protein (g/d)	115 ± 17.0	119 ± 19.3	112 ± 15.5	105 ± 16.6	113 ± 13.8	103 ± 11.7	112 ± 11.5	101 ± 12.5

CG: Control group; SG: Supplement group; TG: Training group; TSG: Training supplement group

### Blood markers

Standardized conditions were used for collecting blood samples that were drawn between 8am and 10am. Blood samples were obtained from the right arm after a 12-hour fast, 48-hour before the initial exercise session and 48 h after the final session. The blood samples were collected in EDTA-containing tubes. Subsequently, a centrifugation process lasting 10 min at 3000 rpm was performed, and the resulting plasma was stored at -80 °C.

Plasma glucose levels were quantified using a colorimetric enzymatic kit (Pars Azmun, Tehran, Iran) with a sensitivity of 5 mg/dl. For insulin level assessment, an ELISA kit (Demeditec, Germany) was used, with a sensitivity of 1 ng/ml and a within-coefficient variations of 5.1–8.4%. Insulin resistance was calculated with the homeostasis model assessment of insulin resistance (HOMA-IR), using the equation: (fasting insulin in μU/mL × fasting glucose in mmol/L)/22.5.

Measurement of plasma lipocalin-2 levels was made using an ELISA kit (Biovendor, Czech Republic; Catalogue No: RD191102200R), having a sensitivity of 0.02 ng/ml. The intra-assay coefficient of variation (CV) was 7.7%, while the inter-assay CV was 9.8%. Plasma omentin-1 levels were quantified utilizing an ELISA kit (Biovendor, Czech Republic; Catalogue No: RD191100200R) having a sensitivity of 0.5 ng/ml, and an intra-assay CV of 3.7%, with an inter-assay CV of 4.6%.

An ELISA kit (Phoenix Pharmaceuticals Inc, USA; Catalogue No: EK 067 − 16) was used to assess plasma irisin levels; the kit had a sensitivity of 6.8 ng/ml. Intra-assay CV was less than 10%, while the inter-assay CV was less than 15%. Plasma spexin levels were measured with an ELISA kit (Elabscience Biotechnology, USA; Catalogue No: E-EL-H5607) with an intra-assay CV of < 10%. Plasma asprosin levels were determined using an ELISA kit (Elabscience Biotechnology, USA; Catalogue No: E-EL-H2266) having an intra-assay CV of < 10%.

### Statistical analysis

Descriptive statistics, presented as means along with standard deviations (SD), described the entirety of the collected data. The Shapiro–Wilk test was used to ascertain the normality of the data distribution. A two-way ANOVA repeated measures test was used to evaluate Group × Time interactions. Baseline data for the four groups underwent assessment using one-way ANOVA and Fisher LSD post hoc tests. In instances where ANOVA detected a significant difference, pairwise comparisons were conducted to ascertain mean differences. The determination of sample size was used to identify a statistical difference in study variables, maintaining a 95% confidence interval (CI) with a power value equal to or exceeding 80%. Additionally, effect sizes were included in the analysis, reported as partial eta-squared (η^2^). Adhering to the classification by Hopkins et al. (2009) [32], effect sizes were categorized as trivial (< 0.2), small (0.2–0.6), moderate (0.6–1.2), large (1.2–2.0), and very large (2.0–4.0). The threshold for statistical significance was set at a *p*-value of < 0.05. All statistical analyses were conducted using SPSS software, version 24 (Chicago, Illinois, USA).

## Results

### Anthropometric indices, and VO_2peak_

The baseline differences between the four groups were not significant for body mass (*p* = 0.46) or BMI (*p* = 0.46). Body mass was different from baseline in the TSG (*p* = 0.039), but not in the SG (*p* = 0.72), TG (*p* = 0.12) or CG groups (*p* = 0.70) (Table 2). Values of BMI were not different from baseline for any of the four groups (*p* > 0.05) (Table 2). There were no significant interactions between group and time for either weight (*p* = 0.28, η^2^ = 0.08) or BMI (*p* = 0.36, η^2^ = 0.07). Values of FFM (*p* = 0.99) and FAT (*p* = 0.33) did not differ in the four groups at baseline. There was an increase in FFM in TSG (*p* = 0.002) but not in SG (*p* = 0.067), TG (*p* = 0.056), or CG (*p* = 0.42). There was an interaction between time and groups for FFM (*p* = 0.046, η^2^ = 0.18). A Bonferroni post hoc test indicated that FFM in the TSG was higher than in CG (*p* = 0.039) (Table 2). There were reductions from baseline FAT in SG (*p* = 0.004), TG (*p* < 0.0001) and TSG (*p* < 0.0001), but not in CG (*p* = 0.28). The time x group interaction for FAT was also significant (*p* < 0.001, η^2^ = 0.46). Fat percentage was higher in CG compared to SG (*p* = 0.036), TG (*p* = 0.0001) and TSG (*p* = 0.0001), while there were no differences between the TSG and SG (*p* = 0.078) and TG (*p* = 0.99) or between the TG and SG (*p* = 0.83) (Table 2).

There were no differences in VO_2peak_ between the four study groups at baseline (*p* = 0.10). There were significant increases in VO_2peak_ from baseline in TG (*p* = 0.001) and TSG (*p* = 0.0001) after 12 weeks, but with no changes in CG (*p* = 0.29) or SG (*p* = 0.15). The interaction between time and groups was significant for VO_2peak_ (*P* = 0.001, η^2^ = 0.35). In comparison to the CG, VO_2peak_ was higher in TG (*p* = 0.003) and TSG (*p* = 0.001) after 12 weeks, but not in SG (*p* = 0.51). There were no differences in VO_2peak_ between the SG and TG (*p* = 0.30) or between the TSG and TG (*p* = 0.99) or SG (*p* = 0.15) (Table 2).

**Table 2:** Mean (± SD) values of glucose, insulin, lipid profile, body composition, and VO_2peak_ in the four study groups

	**CG**			**SG**		TG		**TSG**	
	Pre		Post	Pre	Post	Pre	Post	Pre	Post
Body height (cm)	175.7 ± 4.2	-		171.3 ± 4.2	-	173.3 ± 8.2	-	175.2 ± 6.5	-
Body mass (kg)	101.2 ± 5.3	102.0 ± 2.5		97.8 ± 4.7	97.05 ± 2.45	99.5 ± 10.1	96.2 ± 2.4	101.5 ± 7.9	96.9 ± 1.9 ^&^
BMI (kg/m^2^)	32.8 ± 1.2	33.1 ± 1.4		33.3 ± 0.6	33.13 ± 1.99	33.0 ± 0.8	32.2 ± 2.7	33.0 ± 1.0	31.7 ± 2.2
FFM (kg)	26.4 ± 2.8	25.7 ± 2.8		26.2 ± 2.2	27.86 ± 1.18	26.5 ± 1.8	28.2 ± 2.4	26.4 ± 2.1	29.2 ± 2.3 ^&,*,#^
FAT (kg)	31.1 ± 1.5	31.8 ± 2.1		31.1 ± 1.6	29.08 ± 0.80 ^&,*^	31.4 ± 1.5	27.9 ± 0.9 ^&, *^	32.1 ± 1.4	27.6 ± 1.2 ^&,*,#^
VO_2peak_ (mL⋅kg^− 1^⋅min^− 1^)	26.6 ± 1.8	25.7 ± 1.7		26.7 ± 1.4	27.92 ± 2.32 ^&,*^	26.4 ± 1.3	29.9 ± 2.1^&, *^	26.5 ± 1.8	30.4 ± 1.9 ^&,*,#^
Glucose (mg/dl)	96.4 ± 13.1	98.0 ± 9.4		98.9 ± 10.7	84.77 ± 4.50 ^&,*^	99.3 ± 5.7	74.1 ± 5.44 ^&,*^	101.6 ± 7.1	71.5 ± 7.7 ^&,*,#^
Insulin (ng/ml)	18.8 ± 0.7	19.1 ± 0.6		18.8 ± 0.8	17.60 ± 0.52 ^&,*^	18.8 ± 0.4	15.8 ± 1.1 ^&,*^	19.1 ± 0.5	15.3 ± 0.8 ^&,*,#^
HOMA -IR	4.5 ± 0.7	4.6 ± 0.5		4.6 ± 0.5	3.69 ± 0.25 ^&,*^	4.6 ± 0.3	2.9 ± 0.^3 &,*^	4.8 ± 0.4	2.7 ± 0.3 ^&,*,#^

CG: Control group; SG: Supplement group; TG: training group; TSG: training + Supplement group BMI: Body Mass Index; FFM: Fat-Free Mass; HOMA-IR: Homeostatic Model Assessment of Insulin Resistance

^&^ Significant difference compared to pre-test values (*p* < 0.05)

^*^ Significant difference from the control group (*p* < 0.05)

^#^ Significant interaction between time and groups (*p* < 0.05)

### Glucose, insulin, and HOMA

Baseline values for glucose (*p* = 0.66), insulin (*p* = 0.53), and HOMA-IR (*p* = 0.49) were not different between the groups. The time x group interaction for glucose (*p* = 0.0001, η^2^ = 0.60), insulin (*p* = 0.0001, η^2^ = 0.73) and HOMA-IR (*p* = 0.0001, η^2^ = 0.76) were significant. Reductions in glucose levels in the SG (*p* = 0.0001), TG (*p* = 0.0001), and TSG (*p* = 0.0001) were significant compared to baseline, while there were no changes in CG (*p* = 0.62). Blood glucose was different in SG (*p* = 0.006), TG (*p* = 0.0001) and TSG (*p* = 0.0001) compared to CG. In addition, the reduction in blood glucose in TSG was greater than in SG (*p* = 0.005) but not than in TG (*p* = 0.99). The differences between glucose in the SG and TG were not significant (*p* = 0.10) (Table 1).

There were no differences for insulin at baseline (*p* > 0.05) in the four study groups. Insulin levels decreased after 12 weeks in SG (*p* = 0.0001), TG (*p* = 0.0001) and TSG (*p* = 0.0001) compared to baseline, while there was no change in CG (*p* = 0.31). A Bonferroni test indicated that the reductions in insulin after 12 weeks in SG (*P* = 0.006), TG (*P* = 0.0001) and TSG (*P* = 0.0001) were different from CG. The reduction in the TSG was greater than in SG (*p* = 0.0001) but not compared to TG (*p* = 0.58). The reduction in insulin levels in SG was greater compared to TG (*p* = 0.001) (Table 1***).***

A similar pattern of changes was observed in HOMA-IR. No differences in HOMA-IR were observed between the groups at baseline, but HOMA-IR was lower in SG (*p* = 0.001), TG (*p* = 0.001) and TSG (*p* = 0.001) after 12 weeks, with no change in CG (*p* = 0.36). The reductions in HOMA-IR in SG (*p* = 0.0001), TG (*p* = 0.0001) and TSG (*p* = 0.0001) were different from changes in CG. Reductions in HOMA-IR in TSG (*p* = 0.0001) and TG (*p* = 0.0002) were greater than changes in SG, while there were no differences between TG and TSG (*p* = 0.59) (Table 1).

**Fig. 1:**
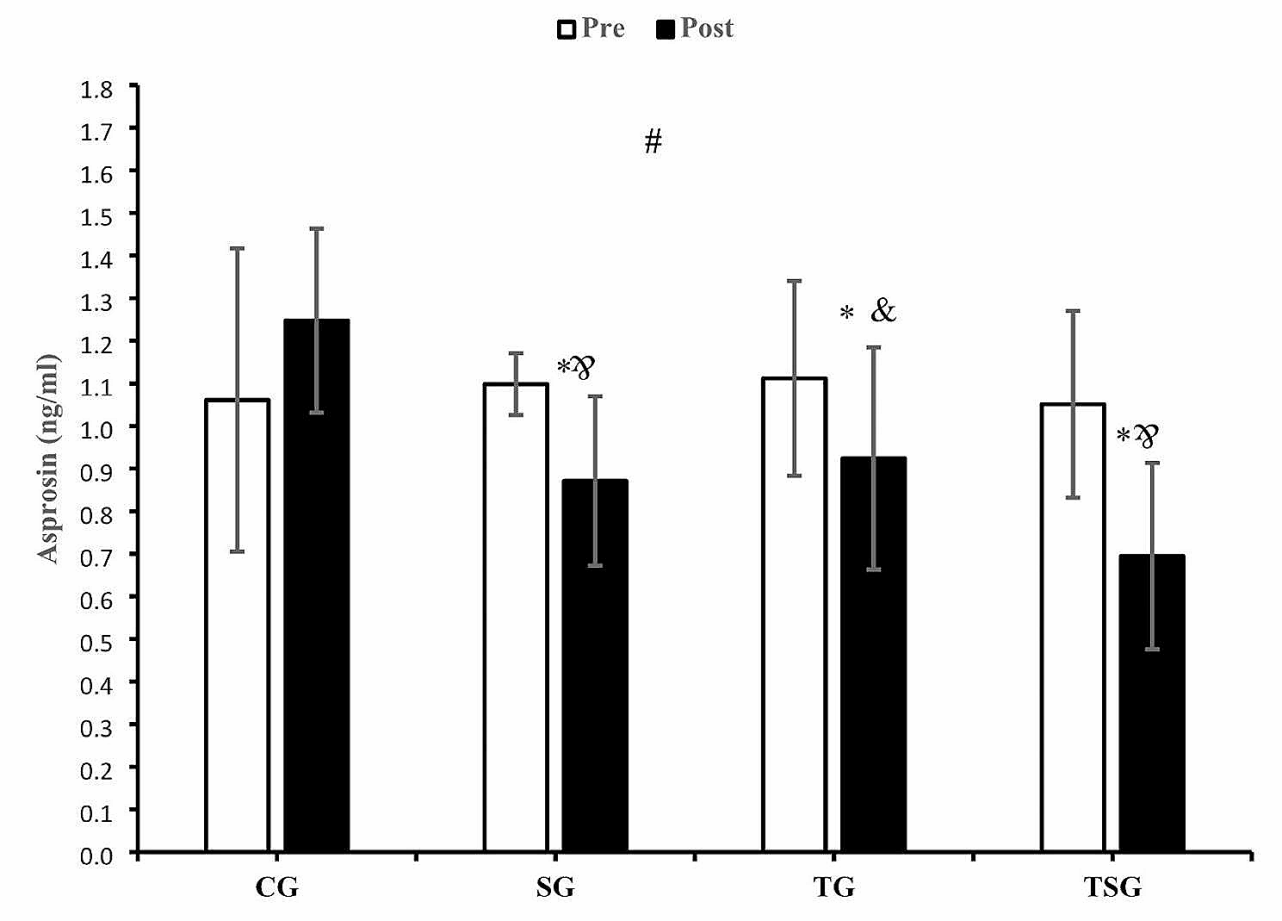
Pre- and post-training values (mean ± SD) for asprosin in control (CG), supplement (SG), training (TG), training + supplement (TSG) groups &: different from pretest values (*p* < 0.05) *: different from control group (*p* < 0.05) #: interaction between time and groups (*p* < 0.05) Correct “&” in TSG bar graphs

There were no differences at baseline between the four groups for asprosin (*p* = 0.92), spexin (*p* = 0.88), lipocalin2 (*p* = 0.89), omentin (*p* = 0.35), or irisin (*p* = 0.71). The results of repeated measures ANOVA indicated a significant interaction between time and groups asprosin (*p* = 0.001, η^2^ = 0.33) (Fig. 1). An analysis using Bonferroni post hoc test indicated that the increases in asprosin after 12 weeks in CG were different from the reduction in SG (*p* = 0.017), TG (*p* = 0.038) and TSG (*p* = 0.001), while the differences between TSG and TG (*p* = 0.99) and SG (*p* = 0.99) and also between TG and SG (*P* = 0.99) for reductions after 12 weeks were not significant. In terms of within-group changes, there were increases in asprosin after 12 weeks in CG (*p* = 0.049) and the decreases in asprosin in SG (*p* = 0.018), TG (*p* = 0.007) and TSG (*p* = 0.047) (Fig. 1).

**Fig. 2:**
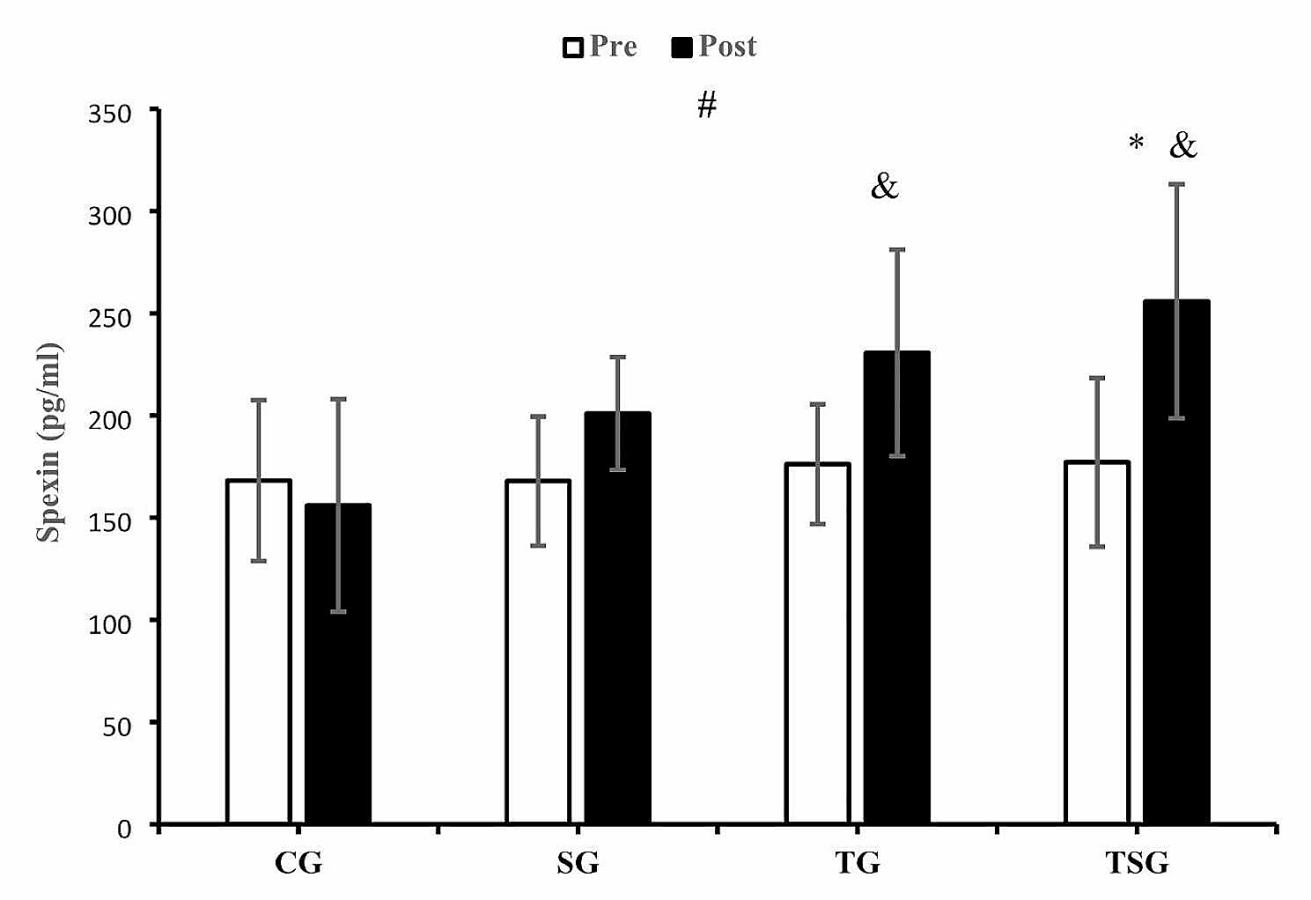
Pre- and post-training values (mean ± SD) for spexin in control (CG), supplement (SG), training (TG), training + supplement (TSG) groups &: different from pretest values (*p* < 0.05) *: different from control group (*p* < 0.05) #: interaction between time and groups (*p* < 0.05)

The interaction between time and groups was significant for spexin (*p* = 0.014, η^2^ = 0.23) (Fig. 2). The post hoc test results indicated that spexin levels only increased after in TSG compared to changes in CG (*p* = 0.011), but other between-group changes were not significant (*p* = 0.99). Pre-post changes in TG (*p* = 0.007) and TSG (*p* = 0.0001) were significant, while the decrease of spexin in CG (*p* = 0.53) and the increase in SG (*p* = 0.095) were not significant (Fig. 2).

**Fig. 3:**
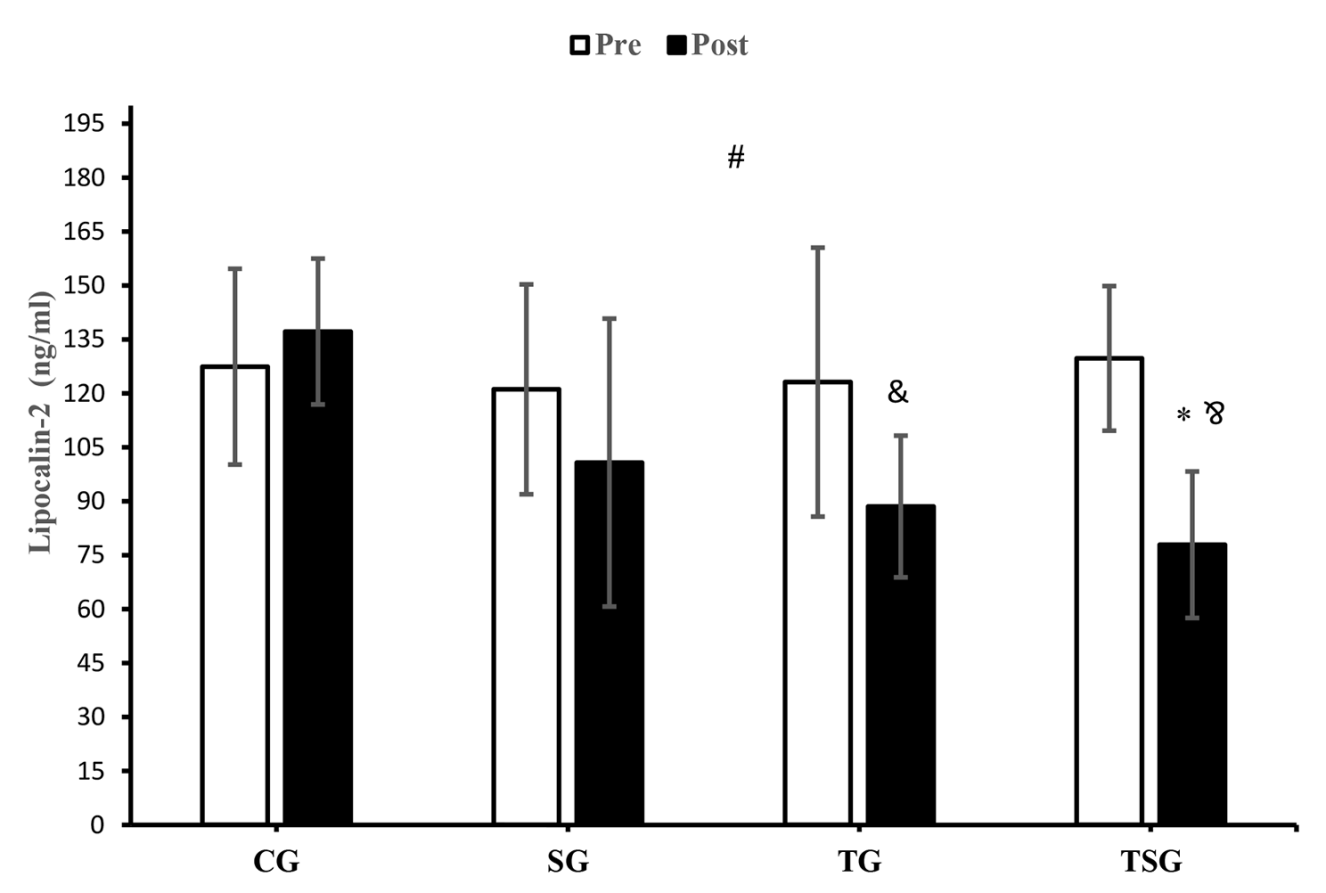
Pre- and post-training values (mean ± SD) for lipocalin-2 in control (CG), supplement (SG), training (TG), training + supplement (TSG) groups &: different from pretest values (*p* < 0.05) *: different from control group (*p* < 0.05) #: interaction between time and groups (*p* < 0.05) Correct “&” in TSG bar graphs

There was a significant interaction between time and groups for lipocalin-2 (*p* = 0.005, η^2^ = 0.27) (Fig. 3). There were differences between decreased lipocalin-2 levels in TSG and the increased level of lipocalin-2 in CG (*p* = 0.004) after 12 weeks, while other between-group changes were not statistically significant (*p* = 0.99). In comparison with pre-test levels, the decreased post-test values of lipocalin-2 in TG (*p* = 0.005) and TSG (*p* = 0.0001) were significant, while the increase of lipocalin-2 in CG (*p* = 0.40) and decreased levels in SG (*p* = 0.08) were not significant (Fig. 3).

**Fig. 4:**
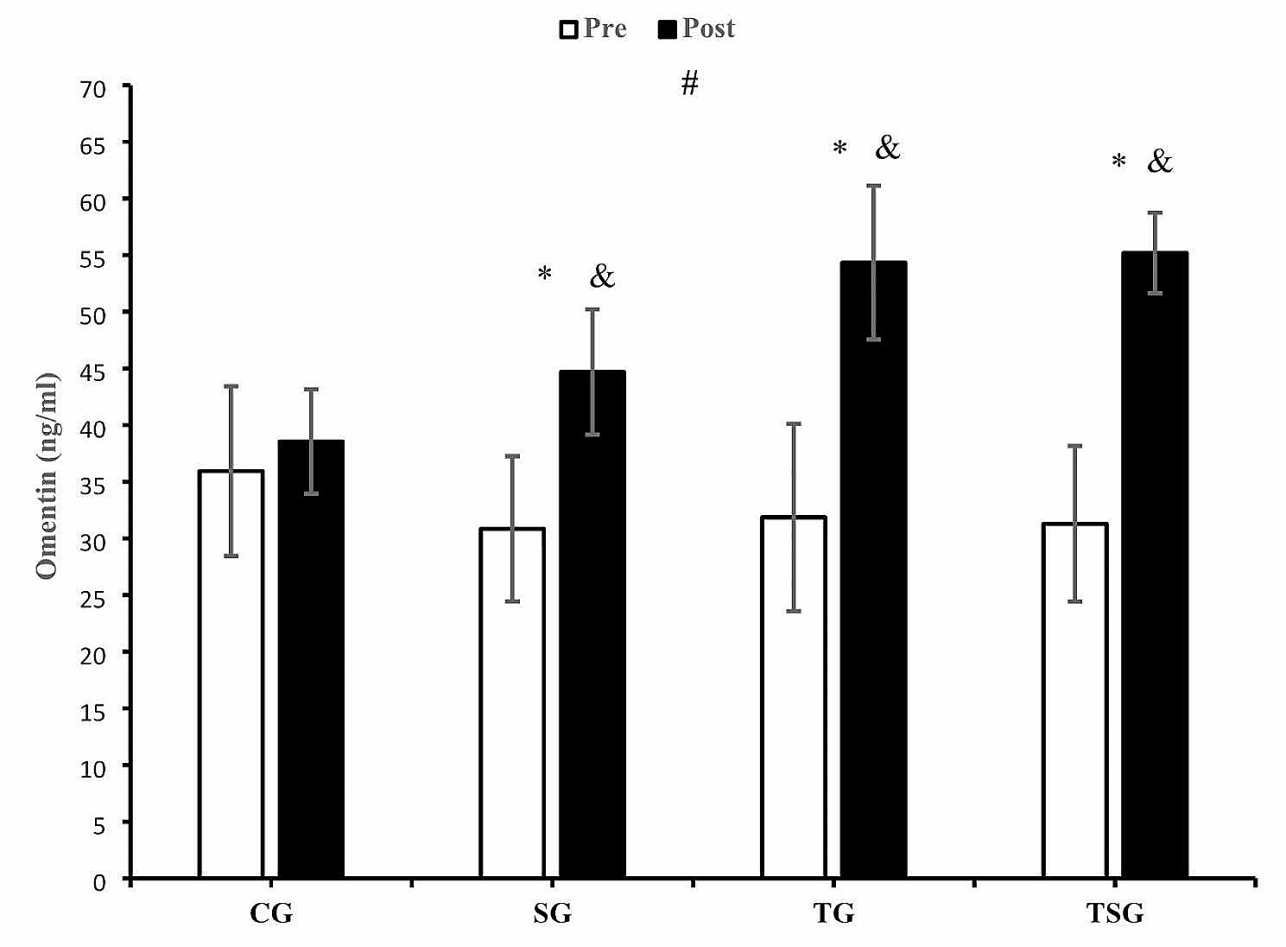
Pre- and post-training values (mean ± SD) for omentin in control (CG), supplement (SG), training (TG), training + supplement (TSG) groups &: different from pretest values (*p* < 0.05) *: different from control group (*p* < 0.05) #: interaction between time and groups (*p* < 0.05)

There were significant time and group interactions for omentin (*p* = 0.0001, η2 = 0.50) (Fig. 4). The increase in CG was lower than in SG (*p* = 0.032), TG (= 0.0001), and TSG) (*p* = 0.0001). Moreover, increases in omentin levels in TG were not greater than in SG (*p* = 0.17). Additionally, increases in omentin levels in TSG did not differ from either TG (*p* = 0.99) or SG (*p* = 0.07). Omentin values increased in SG (*p* = 0.0001), TG (*p* = 0.0001), and TSG (*p* = 0.0001), whereas no change occurred in CG (*p* = 0.34) (Fig. 4).

**Fig. 5:**
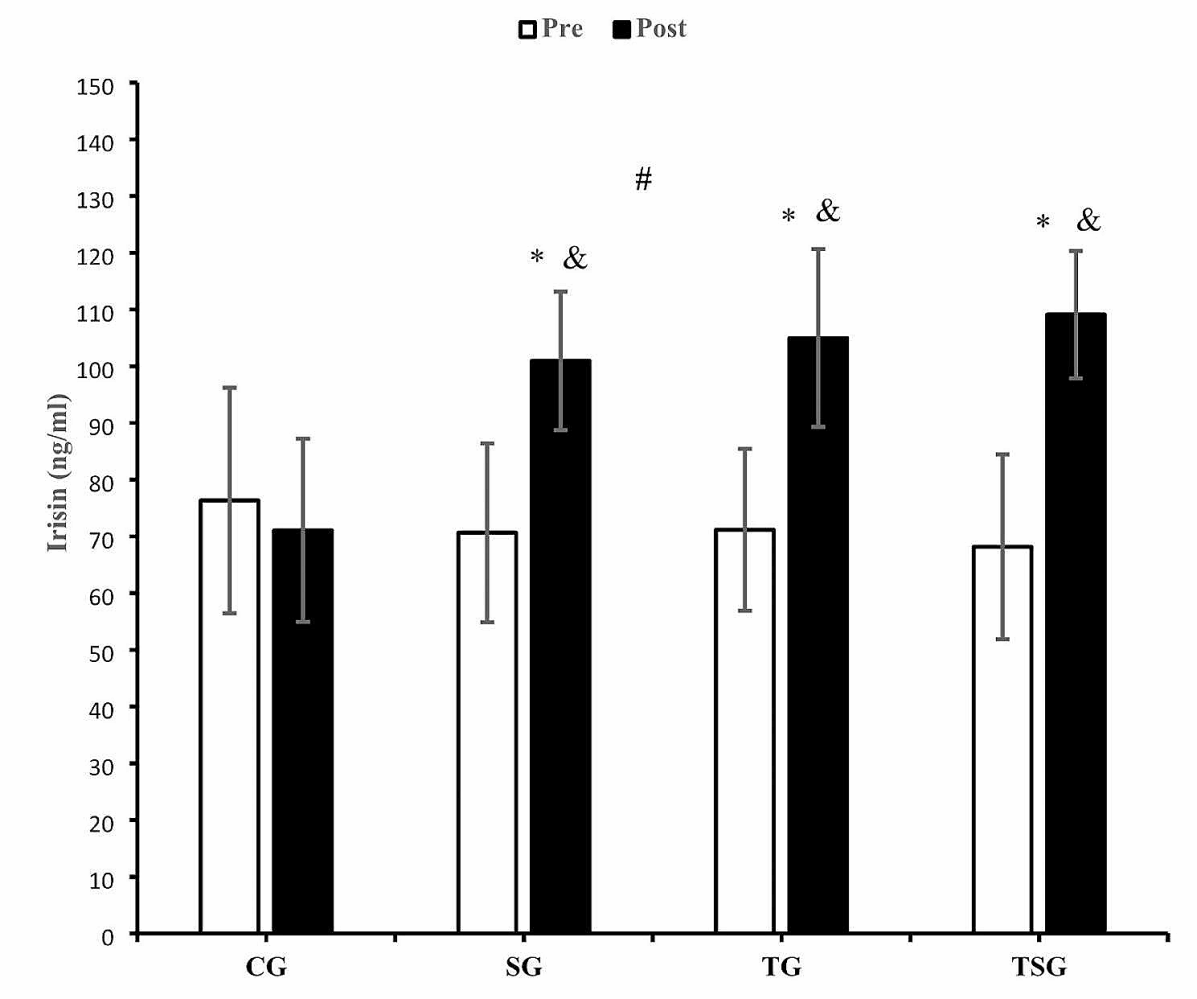
Pre- and post-training values (mean ± SD) for irisin in control (CG), supplement (SG), training (TG), training + supplement (TSG) groups &: different from pretest values (*p* < 0.05) *: different from control group (*p* < 0.05) #: interaction between time and groups (*p* < 0.05)

There was an interaction between time and groups for irisin levels (*p* = 0.0001, η^2^ = 0.39) (Fig. 5). Changes in irisin levels in CG after 12 weeks were different from the increased levels of irisin in SG (*p* = 0.006), TG (*p* = 0.002) and TSG (*p* = 0.0001). The increase in irisin levels in TG were not higher than in SG (*p* = 0.99). Irisin levels in TSG were higher than in TG (*p* = 0.99) or CG (*p* = 0.99). The reduction in irisin levels in CG after 12 weeks was different from pretest values (*p* = 0.46). There were increases in irisin levels in SG (*p* = 0.0001), TG (*p* = 0.0001) and TSG (*p* = 0.0001) after 12 weeks (Fig. 5).

## Discussion

This study indicates that a 12-week intervention of HIIT and spirulina supplementation, individually or in combination, improves the levels of some circulating adipokines in obese men. Notably, the combined intervention of HIIT and spirulina yielded greater improvements in the measured outcomes compared with each intervention alone. Specifically, we report reductions in plasma lipocalin-2 and asprosin levels combined with increases in irisin, omentin, and spexin levels produced by HIIT and spirulina supplementation. Furthermore, this combined approach increased VO_2peak_ and FFM while decreasing body fat levels and insulin resistance.

### Anthropometric indices and cardiorespiratory parameters

Our findings provide insights into the intricate relationship between spirulina supplementation, HIIT, and body composition, with a particular focus on the interplay of these factors on fat percentage. The combined use of spirulina and HIIT enhanced FFM and simultaneous FAT reductions. Notably, HIIT alone induced changes specifically in FAT, while sole supplementation with spirulina failed to elicit modifications in either FFM or FAT. This observation suggests a synergistic interaction between spirulina’s antioxidant effects and the physiological benefits of HIIT, which leads to nuanced alterations in body composition.

The reductions in FAT can be reasonably attributed to the greater energy expenditure associated with HIIT. Despite not providing daily energy expenditure details, our analysis indicated no energy intake discrepancies between pre- and post-intervention reporting periods. This alignment with our results corroborates findings from previous studies showcasing the efficacy of a 12-week HIIT regimen leading to decreases in abdominal, trunk, and visceral fat in overweight young males [32, 33].

Furthermore, the increase in FFM in the combined HIIT and supplement group in our study aligns with previous reports that underscored spirulina’s benefits on lean body mass and body composition, particularly in middle-aged and elderly individuals [24, 35]. Additionally, prior studies highlighted spirulina’s potential to enhance body fat percentage, reduce fat mass, and modulate myostatin, follistatin, and insulin-like growth factor 1 (IGF-1) levels, factors that are pivotal in regulating muscle mass [24, 35]. Our findings align with this body of work, suggesting spirulina’s positive influence on lean body mass in the context of training and weight loss regimens.

The preservation or promotion of muscle mass has important implications for metabolic health by fending off an array of obesity-associated ailments, such as insulin resistance, type 2 diabetes, and cardiovascular disorders [36–38]. Likewise, there is much evidence to support a link between cardiorespiratory fitness, cardiometabolic health, and all-cause mortality [39]. While our study demonstrated a boost in VO_2peak_ due to HIIT, it is important to note that the incorporation of spirulina failed to yield further increases in VO_2peak_ compared to HIIT alone. The existing literature describing spirulina’s impact on aerobic exercise performance remains inconclusive, with conflicting evidence proposing both ergogenic and non-ergogenic effects [40].

### Asprosin

Asprosin, a glucogenic adipokine secreted by white adipocytes, participates in numerous physiological processes, including hepatic glucose production and modulation of metabolic dysfunction in obesity [41, 42]. The impact of asprosin on obesity remains unclear, but studies suggest its involvement in the early stages of dysglycemia and obesity-related disorders [41]. Notably, aerobic exercise reduces hepatic asprosin expression, indicating a potential link between exercise and asprosin regulation [42].

Our 12-week study involving HIIT, with or without daily spirulina supplementation, reduced circulating asprosin levels in obese males, a reduction that was also observed with spirulina supplementation alone. Both exercise and spirulina supplementation enhance antioxidant capacity, reduce oxidative stress, and decrease markers of inflammation [42–45]. Our findings suggest that the changes in asprosin levels may be associated with reduced oxidative stress in white adipocytes, potentially leading to an improved proinflammatory secretory profile [46, 47]. Importantly, our study highlights the potential synergistic effects of spirulina and HIIT in decreasing asprosin levels, contributing to improved blood glucose, insulin levels, and increased insulin sensitivity in obesity. Further exploration and large-scale clinical studies are warranted to validate these effects and understand the underlying mechanisms.

### Spexin

Spexin, a recently identified secretion from adipose tissue, plays a pivotal role in regulating energy homeostasis and adipocyte function. Levels of spexin are decreased in adipocytes and also in the circulation in obesity [15]. Emerging evidence from animal studies suggests that spexin protects against obesity by impeding adipogenesis, modulating the expression of pro-adipogenic genes [48], reducing weight and improving insulin sensitivity [49]. Notably, spexin is believed to participate in counteracting obesity through mechanisms involving appetite suppression and enhanced physical activity [50, 51].

Human studies indicate that circulating spexin levels are reduced in obesity, exhibiting a negative correlation with multiple markers such as leptin, ghrelin, total cholesterol, LDL, blood pressure, insulin, HOMA-IR, BMI, and waist-to-hip ratio [15]. Conversely, there are also positive correlations between adiponectin, glucagon-like peptide 1, and insulin sensitivity [48, 52]. Intriguingly, obese subjects undergoing a combination of aerobic and resistance training have increases in spexin levels, mostly in individuals with maximal oxygen consumption, C-reactive protein, total cholesterol, HbA1c, and HOMA-IR, underscoring the potential of spexin as a biomarker for obesity and its related cardiometabolic manifestations [15].

Our study reports increases in circulating spexin levels after 12 weeks of HIIT independent of supplementation with spirulina. To the best of our knowledge, our investigation is the first to report a beneficial effect of HIIT on circulating spexin levels. This phenomenon is presumed to contribute to the favorable health outcomes associated with HIIT in obese individuals.

### Lipocalin-2

The peptide lipocalin-2 is expressed in various tissues, including adipose tissue, and is released into circulation as an adipokine [53], and plays a role in metabolic dysregulation associated with obesity and diabetes; however, its precise function remains unclear [54]. For instance, some studies suggest that lipocalin-2 reduces food intake, fat mass, and body weight gain in rodent, while simultaneously enhancing glucose metabolism [54, 55]. Furthermore, lipocalin-2 may have protective effects against obesity-related metabolic impairments, fatty liver disease, atherogenic dyslipidemia, insulin resistance, and is also associated with the modulation of hepatic gluconeogenesis, adaptive thermogenesis, activation of brown fat, and fatty acid oxidation [56–59].

Reports on the impact of exercise on lipocalin-2 levels are inconsistent; some studies reported no changes after concurrent training or endurance/resistance training [60, 61], while other studies report either increased lipocalin-2 levels following 12 weeks of HIIT in obese males [62] or observed decreases with endurance and resistance training in sedentary young men [63]. Our study, involving 12 weeks of HIIT in obese males, demonstrated a reduction in lipocalin-2 levels. Discrepancies in findings may be attributed to differences in HIIT protocols, including work interval durations and overall exercise duration. Notably, spirulina supplementation did not enhance lipocalin-2 levels during HIIT or as a standalone supplement.

### Omentin-1

Our investigation provides intriguing insights into the modulation of omentin-1 levels, which diverge from our prior study outcomes indicating that 12 weeks of HIIT reduced omentin-1 levels in obese males [63], whereas our study suggests incrreases in omentin-1 levels both in the presence and absence of spirulina supplementation. Notably, spirulina supplementation in isolation increased omentin-1 levels, corroborating similar observations where plasma omentin-1 concentrations were increased after four weeks of HIIT, regardless of spirulina incorporation [24].

Findings on the impact of exercise on circulating omentin-1 levels remain intricate and heterogeneous, with a spectrum of findings, where some investigations reporting heightened omentin-1 levels due to exercise [64], while others observed decreases [65, 66]. This variability may be due to multifaceted factors inherent to the populations and methodologies employed.

Circulating omentin-1 levels, often attenuated in individuals grappling with obesity, serve as a potential biomarker for metabolic risk, given their inverse correlation with cardiometabolic risk factors [17, 67, 68]. The intricate interplay between omentin-1 expression, plasma concentrations, and visceral adipose tissue is underscored by diminished omentin-1 expression in visceral adipocytes in those afflicted by obesity [67]. Emerging evidence suggests that omentin-1 has beneficial effects on insulin sensitivity, affording protection against atherogenesis through its involvement in macrophage differentiation, inflammation, arterial calcification, and plaque formation [68]. Furthermore, decreased omentin-1 concentrations correlate with elevated carotid intima-media thickness in healthy males [68] and are implicated in coronary artery disease [69, 70]. These beneficial metabolic and atherogenic effects are postulated to emanate from the inherent anti-inflammatory and antioxidant properties of omentin-1 [68, 71]. While the precise impact of exercise, particularly HIIT, on omentin-1, remains to be elucidated, the decreases in circulating omentin-1 levels potentially signify improvements in metabolic and cardiovascular health, accompanied by reduced disease susceptibility.

### Irisin

The adipokine irisin improves metabolic functionality induced by exercise in various tissues such as skeletal muscle, adipose tissue, the pancreas, liver, bone, the central nervous system, and the endothelium. Exercise augments PCG1-α expression in skeletal muscle, and triggers the synthesis of fibronectin type III domain-containing protein 5 (FNDC5) which is secreted into the bloodstream after cleavage [72]. Notably, irisin improves glucose and lipid metabolism, mitigating the impacts of obesity-related inflammation, metabolic syndrome, and diabetes [73, 74].

Our study indicates that 12 weeks of HIIT, either coupled with daily spirulina supplementation or pursued independently, leads to an elevation in circulating irisin levels among obese males. Other studies report increases in in irisin concentrations following high-intensity exercise, albeit with a limited focus on the implications of HIIT [72, 75, 76]. A separate study by Murawska-Cialowicz et al. observed heightened irisin levels in non-obese, prediabetic men after an eight-week HIIT regimen [76]. To our knowledge, there is a dearth of data on the impact of dietary antioxidant supplementation, such as that involving spirulina, on the modulation of circulating irisin levels.

The therapeutic potential of irisin extends across various health domains, including obesity, type 2 diabetes, cardiovascular ailments, stroke, neurodegenerative disorders, cancer, and sarcopenia [77–80], underscoring the notion that the amalgamation of HIIT and spirulina supplementation-induced elevations in irisin levels may furnish protection against the adverse consequences associated with obesity.

### Study limitations

Our study has several limitations that warrant consideration: [1] The exclusive inclusion of male participants with obesity raises questions about the generalizability of our findings, particularly to females and individuals with varied demographic profiles; [2] The emphasis on blood levels of adipokines may limit insights into tissue-specific changes. To provide a more comprehensive understanding, future research should consider incorporating additional factors, such as a detailed exploration of oxidative stress and energy expenditure, to further contextualize our findings.

## Conclusions

Our findings contribute to the expanding body of evidence that underscores the potential of HIIT in ameliorate adipokine profiles in the context of obesity. Moreover, our study suggests that supplementation with spirulina may be a promising approach for diminishing pro-inflammatory adipokines while concurrently enhancing anti-inflammatory adipokines. This holds significance due to emerging indications of the important role of adipokines in mediating the metabolic, cardiovascular, and inflammatory imbalances inherent in obesity. Their secretory composition is implicated in either exacerbating or safeguarding against a spectrum of obesity-associated disorders. Further investigations are needed to establish more robustly the impact of therapeutic interventions using HIIT and dietary antioxidants on refining adipokine profiles in obesity.

## Data Availability

No datasets were generated or analysed during the current study.
